# Too poor or too far? Partitioning the variability of hospital-based childbirth by poverty and travel time in Kenya, Malawi, Nigeria and Tanzania

**DOI:** 10.1186/s12939-020-1123-y

**Published:** 2020-01-28

**Authors:** Kerry L. M. Wong, Oliver J. Brady, Oona M. R. Campbell, Aduragbemi Banke-Thomas, Lenka Benova

**Affiliations:** 1 Department of Infectious Disease Epidemiology, Faculty of Epidemiology and Population Health, London School of Hygiene and Tropical Medicine, Keppel Street, London, WC1E 7HT UK; 2 Centre for Mathematical Modelling for Infectious Diseases, London School of Hygiene and Tropical Medicine, Keppel Street, London, WC1E 7HT UK; 3 Department of Health Policy, London School of Economics and Political Science, Houghton Street, London, WC2A 2AE UK; 4 Department of Public Health, Institute of Tropical Medicine, Kronenburgstraat 43, 2000 Antwerp, Belgium

**Keywords:** Health equity, Maternal health, Low- and middle-income countries, Wealth inequality, Physical accessibility, Hospital-based childbirth, Skilled care at birth, Pregnancy and childbirth

## Abstract

**Background:**

In sub-Saharan Africa, women are most likely to receive skilled and adequate childbirth care in hospital settings, yet the use of hospital for childbirth is low and inequitable. The poorest and those living furthest away from a hospital are most affected. But the relative contribution of poverty and travel time is convoluted, since hospitals are often located in wealthier urban places and are scarcer in poorer remote area. This study aims to partition the variability in hospital-based childbirth by poverty and travel time in four sub-Saharan African countries.

**Methods:**

We used data from the most recent Demographic and Health Survey in Kenya, Malawi, Nigeria and Tanzania. For each country, geographic coordinates of survey clusters, the master list of hospital locations and a high-resolution map of land surface friction were used to estimate travel time from each DHS cluster to the nearest hospital with a shortest-path algorithm. We quantified and compared the predicted probabilities of hospital-based childbirth resulting from one standard deviation (SD) change around the mean for different model predictors.

**Results:**

The mean travel time to the nearest hospital, in minutes, was 27 (Kenya), 31 (Malawi), 25 (Nigeria) and 62 (Tanzania). In Kenya, a change of 1SD in wealth led to a 33.2 percentage points change in the probability of hospital birth, whereas a 1SD change in travel time led to a change of 16.6 percentage points. The marginal effect of 1SD change in wealth was weaker than that of travel time in Malawi (13.1 vs. 34.0 percentage points) and Tanzania (20.4 vs. 33.7 percentage points). In Nigeria, the two were similar (22.3 vs. 24.8 percentage points) but their additive effect was twice stronger (44.6 percentage points) than the separate effects. Random effects from survey clusters also explained substantial variability in hospital-based childbirth in all countries, indicating other unobserved local factors at play.

**Conclusions:**

Both poverty and long travel time are important determinants of hospital birth, although they vary in the extent to which they influence whether women give birth in a hospital within and across countries. This suggests that different strategies are needed to effectively enable poor women and women living in remote areas to gain access to skilled and adequate care for childbirth.

## Background

Ensuring skilled care at birth, with the right person in an enabled environment, can prevent mortality and morbidity in women and newborns. In high-burden and resource-scarce settings, such as countries of sub-Saharan Africa, the use of skilled care at birth is still far from universal [1]. A wide range of different social, woman, birth-related, and macro-level barriers to using skilled care at birth have been identified in the literature [2–4]. Low household wealth/socioeconomic status (SES) and problematic physical accessibility to an adequate provider are amongst the most persistent barriers. A number of studies have shown that wealthier women consistently report higher use of skilled care at childbirth than their poorer counterparts [5–7]. For the poor, the direct (e.g. medical bills) and indirect (e.g. transportation, lost earnings) costs associated with seeking and using skilled childbirth care may be unaffordable [8, 9].

In addition to financial affordability, lack of physical accessibility to health services also imposes barriers to using skilled care at birth. Physical accessibility is determined by one’s geographic location, and is captured by factors such as the distribution of facilities, travel time or distance from home to facility, availability of transportation, and the condition of roads. It shapes people’s options for care-seeking and their decision making [10], and can cause delays in reaching an adequate provider when needs arise [3]. The negative effect of poor physical accessibility on the use of skilled care at birth was first reviewed by Thaddeus and Maine in 1994 [4], and reaffirmed in systematic reviews, including Gabrysch and Campbell 2009 [3], Moyer and Mustafa 2013 [2], Wong et al. 2017 [11] and Tegegne et al. 2018 [12].

Removing financial and accessibility barriers may be complicated by the correlation between them [13], since resource and infrastructure often concentrate in wealthier urban places, and are scant in poorer and remote areas. Higher availability and better accessibility to healthcare in wealthier urban places may exacerbate the inequity gap in health service uptake between people living in such places and their counterparts in poorer and remote areas. A recent study of wealth inequalities in travel time to the nearest hospital in Kenya, Malawi, Nigeria and Tanzania found dramatic differences between wealth subgroups. Average travel time to the nearest hospital for the wealthiest decile was < 15 min – 4-14 times shorter compared to the poorest deciles in these countries [14]. Such gap in travel time raises questions regarding the potential overlap of the negative effects of poverty and travel time on use of skilled care at birth, in other words – are women too poor or too far to use skilled care at birth? This question exposes a gap in the current literature about the separate and combined contributions of these two barriers.

To address this question, we propose to examine the variability in the proportion of births occurring in hospitals (rather than in any health facility), since the full range of live-saving “skilled” childbirth services, such as caesarean section and blood transfusion, are typically only available in hospital settings if at all [15]; and equipment and staffing at lower-level, primary facilities (e.g., health centres/posts/huts and dispensaries) are often inadequate for the basic functions that they are expected to provide [16–18]. In this study, we quantify the relative contribution of poverty and travel time on rates of hospital birth in sub-Saharan African countries. We also aim to test if poverty and travel time interact. Our results generate insights that can be used for health policy making to ensure that the most left behind expectant mothers receive skilled and adequate care for childbirth.

## Data and methods

### Study settings

We studied four LMICs in sub-Saharan Africa – Kenya, Malawi (excluding Likoma Island), Nigeria and Tanzania (excluding Zanzibar). These countries were selected over others in the sub-Saharan African region because they had a recent complete list of hospitals with geographic coordinates, and represented different contexts in terms of demography, geography, travel time to the nearest emergency care and facility-based childbirth. National statistics according to the World Bank [19], the Demographic and Health Surveys Program [20], and the 2015 geocoded inventory of emergency hospitals in sub-Saharan Africa by Ouma and colleagues [21] are presented in Table 1.

**Table 1:** Country data and statistics

	Kenya	Malawi	Nigeria	Tanzania
Total area (km^2^) [19]	580,367	118,484	923,768	947,300
National population in 2015 (million) [19]	47	18	181	54
% urban population in 2015 [19]	26	16	48	32
% of all births in health facilities^a^ [20],	61.2	91.4	35.8	62.6
% population > 2 h travel time to public emergency hospital care [21]	7	7	8	25

^a^ The most recent Demographic and Health Survey as of January 2019 for each country – Kenya 2014, Malawi 2015/16, Nigeria 2013 and Tanzania 2015/16

### Data and measurement

We used four data sources: (a) Demographic and Health Surveys (DHS) to determine place of childbirth, household location, household wealth and other potential confounders, (b) a master list of all health facilities with geographic coordinates for each country, (c) the Global Friction Surface 2015 by the Malaria Atlas Project (MAP) is used in conjunction with (a) and (b) to determine travel time from household to hospital, and (d) country administrative boundary files (version 2.5, July 2015) downloaded from the GADM database on gadm.org [22].

First, we used the most recent DHS as of January 2019 for each study country – Kenya 2014, Malawi 2015/16, Nigeria 2013 and Tanzania 2015/16. The DHS collect nationally representative data on population health and sociodemographic characteristics using a multi-stage cluster sampling design with enumeration area as the cluster, or primary sampling unit. As part of the DHS sampling procedure, a list of established households in each sampled cluster is obtained and used as the sampling frame for household selection [23]. All women aged 15–49 in selected households were interviewed with a standardized questionnaire with questions on all their livebirths in the 5 years before the survey. All these births were considered in the current analysis.

In each survey, a household wealth index was constructed by the DHS using household asset data via a principal component analysis [24]. Each livebirth is assigned its household’s wealth index. The outcome of interest is hospital-based childbirth. For each livebirth, place of childbirth was based on women’s answer to: “Where did you give birth to [name of child]?” in the Women’s Questionnaire. The major categories of response options were domestic environments (home of respondent, family member, or traditional birth assistant (TBA)), public/government sector health facilities and private/non-government sector health facilities. The DHS conflated clinics and hospitals as one response option for health facilities in the non-government sector for Kenya, Malawi and Nigeria. In line with the approach taken by Hanson and colleagues [25], the categorisation of facility delivery locations into hospital was done in consideration of the local context and health system in each country, and the response options on the survey. Data on other potential predictors of hospital birth, including maternal education, maternal age at birth and birth order, were also sourced from the DHS. We captured the context-specific barriers associated with the lived environment beyond the predictor variables described here by including a random effect at the level of survey cluster.

The DHS include the longitude and latitude coordinates of the population centroids of sampled clusters. All individuals residing in the same cluster have the same geo-referenced location. For anonymity reasons, urban clusters are displaced up to 2 km and rural clusters up to 5 km [26]. We excluded nine clusters in Kenya and seven clusters in Nigeria with missing coordinates from our analysis.

Second, master lists of health facilities were obtained online [27–31]. These lists are inventories of all government and non-government health facilities in the country, with data on facility type – hospital vs. others – and geographic coordinates. These lists contain facility data from 2015 (Kenya), 2013 (Malawi), 2010–2014 (Nigeria) and 2016 (Tanzania).

Third, we quantified physical access as the travel time required to travel from the displaced cluster centroid to the nearest hospital using the MAP Global Friction Surface (the friction surface below) 2015. The friction value represents the generalized difficulty to cross a pixel depending on land surface condition, such as the type of road, water bodies, and terrain with slope. Travel time to the nearest hospital was computed for every 1 × 1 km^2^ pixels covering the study region using an algorithm devised by Weiss and colleagues [32]. This algorithm identifies the path that requires the least time through the friction surface between two points [32], and has been used to construct accessibility maps enumerating travel time to the nearest hospital in previous studies [14, 33]. DHS suggests generating average values using neighbourhood buffers to moderate the potential impact of point displacements [34]. In this study, we extracted travel time values for each DHS cluster as the average of the four nearest pixels.

### Statistical analysis

We tested travel time estimated from the MAP friction surface by comparing 20% of DHS clusters (selected at random) against travel time estimates obtained using data from the OpenStreetMap (OSM) project [35]. We used Pearson correlation coefficient to assess the linear correlation between the two sets of values.

Generalized additive models (GAMs) were used to assess the effects of wealth, travel time to the nearest hospital and other predictor variables on hospital birth [36]. The “mgcv” package for the R statistical package [37] was used to construct mixed-effects GAM models with the application of survey sampling weights. A different GAM was constructed for each country. A GAM model is expressed as

$$ {\displaystyle \begin{array}{c} logit\left( hospital\ birth\right)={f}_1\left( wealth\ index, travel\ time\right)+{f}_2\left( maternal\  age\  at\  birth\right)+\\ {} maternal\ education+ birth\ order\end{array}} $$

We used the logit link logit(*.)* to relate the predictors with the expected value of the response. Smoothing functions *f*_*i*_ are found for the different predictor variables. We tested whether the effect of travel time varied by wealth using an interaction term specified as a scale invariant tensor product smooth. For this term, we tested two different numbers of knots for smoothing – 5 and 10. A penalized thin plate regression spline was fitted to maternal age at birth, as very young and very old women may use hospital childbirth care differently [38]. A truncated eigen-decomposition is used to achieve the rank reduction [37]. Linear terms were used for maternal education and birth order. We applied survey-specific weighting to account for the sampling procedures used in the surveys.

We present the marginal effects of all predictors from the fully-adjusted mixed-effects GAMs. For each model predictor, we calculated the predicted probabilities of hospital birth for every standard deviation (SD) change from mean – μ ± 1SD – whilst holding other predictors at the respective sample mean. These predictions showed the effect that varying each predictor variable within a country’s population would result in. For normally-distributed data, with a mean and median being the same and 68% of the data falling within 1SD from the mean value, the comparison between μ-1SD, μ, μ + 1SD is equivalent to comparing the 16th, 50th and 84th percentiles. The marginal effect of the survey cluster random effect was obtained from the distribution of predicted values with all model predictor variables set to the sample mean. Again, we calculated the predicted probabilities of 1SD around the model mean predicted probabilities of hospital birth.

We further used a response surface to show the additive effect of DHS wealth index and travel time on hospital birth. The predicted probabilities were represented by a colour gradient. Model residuals were plotted as heat maps to show the locations at which the variability of hospital birth was well explained by the fully-adjusted mixed-effects GAM models.

### Ethics approval

The DHS receive government permission and follow ethical practices including informed consent and assurance of confidentiality. The authors requested and received approval to download and use the data from the DHS websites as detailed under the data sharing page. Master facility lists were publicly available [23]. The Research Ethics Committee of the London School of Hygiene and Tropical Medicine approved our secondary-data analysis.

## Results

### Descriptive

**Fig. 1:**
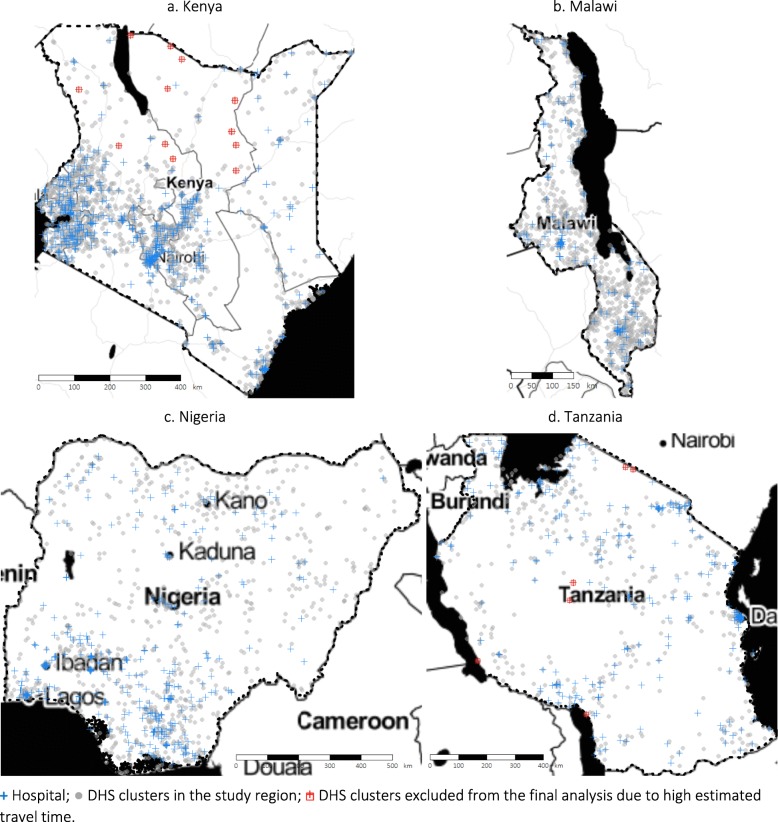
Map of the study region, hospitals and DHS clusters. Hospital;  DHS clusters in the study region; DHS clusters excluded from the final analysis due to high estimated travel time

Across the study countries, the numbers of DHS clusters identified were 1565 (Kenya), 828 (Malawi), 889 (Nigeria), and 527 (Tanzania). Travel time estimated from the MAP friction surface and that obtained using OSM data showed good alignment (Pearson correlation coefficients over 0.75 in all countries, see Additional file 1), apart from a few clusters with long travel time of ≥5 h estimated using the MAP friction surface. For this reason, we excluded 12 and 6 clusters from Kenya and Tanzania from the final analysis (Fig. 1).

The numbers of DHS clusters, livebirths and hospitals used in our final analysis are shown in Table 2, together with summary statistics of travel time to the nearest hospital and the percentage of births in hospitals by country. Overall, Kenya and Nigeria had the shortest mean travel time from clusters to the nearest hospital (about 25 min), and Tanzania the longest (62 min). Travel time was highly right-skewed, and a cube-root transformation was used in subsequent analyses. The percentage of births in hospitals ranged between 27% in Nigeria to 39% in Kenya. Majority of hospital births occurred in government hospitals, except in Nigeria, where the shares of government hospital births and non-government hospital births were similar (Table 2).

**Table 2:** Summary statistics in study countries

		Kenya	Malawi	Nigeria	Tanzania
DHS survey year		2014	2015/16	2013	2015/16
Number of DHS clusters		1585	828	889	527
Number of DHS clusters^a^<5 h from a hospital		1573	828	889	521
Number of livebirths included in the final analysis^b^		19,463	17,384	31,828	8317
Year of master facility list data		2015	2013	2010–2014	2016
Number of hospitals in the master facility list		485	116	3787	265
Number of geo-referenced hospitals		480	115	3787	265
Travel time to the nearest hospital in minutes					
Mean (standard deviation)		26.6 (40.5)	30.9 (28.5)	25.2 (33.5)	61.7 (58.4)
Median (interquartile range)		12.7 (4.1–29.8)	24.9 (10.7–40.7)	14.2 (3.7–34.1)	45.1 (16.9–87.9)
Maximum		291.2	268.3	293.9	296.0
Percentage distribution of place of childbirth among livebirths included in the final analysis^b^					
Hospital	Government sector	30.3	27.4	14.1	23.0
	Non-government sector	9.1	7.9	13.0	8.3
Other health facilities	Government sector	15.8	51.4	8.5	27.1
	Non-government sector	6.1	4.8	0.2	3.6
Not in a health facility (own/TBA/other home)		37.2	7.1	63.2	37.9
Unknown/missing		1.5	1.5	1.0	0.0
Total percentage of hospital childbirth		39.4	35.3	27.1	31.4
Total percentage of facility childbirth		61.3	91.4	35.8	62.1

*TBA* Traditional birth attendant

^a^ Excluding Likoma Island in Malawi (22 DHS clusters) and Zanzibar in Tanzania (81 DHS clusters), and DHS clusters without geographic coordinates (9 in Kenya and 7 in Nigeria)

^b^ The final analysis comprised livebirths from geo-referenced survey clusters < 5 h from a hospital, and with the same residence at the time of survey and birth (where data was available)

### The association of wealth, travel time, and other covariates with hospital birth

The deviances explained by the fully-adjusted mixed-effects GAMs were similar using both 5 and 10 knots for smoothing on the interaction term between travel time and wealth (Additional file 1). We present results from the simpler models with 5 knots. Results of the fully-adjusted mixed-effects GAMs are shown in Table 3. All predictor variables were significant. The mean predicted probabilities of hospital birth obtained from these models were 33.2% (Kenya), 32.7% (Malawi), 26.6% (Nigeria) and 29.6% (Tanzania).

**Table 3:** Results of generalized additive models of hospital-based childbirth by country

	Kenya			Malawi			Nigeria			Tanzania		
Approximate significance of smooth terms	EDF	REF DF	*p*-value	EDF	REF DF	*p*-value	EDF	REF DF	*p*-value	EDF	REF DF	*p*-value
Wealth index × travel time ($\sqrt[3]{\mathrm{hours}}$)	6.48	7.31	< 0.001	10.71	24.00	< 0.001	11.77	24.00	< 0.001	8.37	24.00	< 0.001
Maternal age at birth (years)	2.36	2.96	< 0.001	2.89	9.00	< 0.001	2.54	9.00	< 0.001	3.79	6.00	< 0.001
Parametric coefficients of linear terms	EST	SE	*p*-value	EST	SE	*p*-value	EST	SE	*p*-value	EST	SE	*p*-value
Maternal education (years)	0.06	0.01	< 0.001	0.03	0.01	< 0.001	0.09	0.00	< 0.001	−0.05	0.01	< 0.001
Birth order	− 0.28	0.02	< 0.001	− 0.12	0.02	< 0.001	−0.10	0.01	< 0.001	−0.16	0.03	< 0.001
Random effects	EDF	REF DF	*p*-value	EDF	REF DF	*p*-value	EDF	REF DF	*p*-value	EDF	REF DF	*p*-value
Survey cluster	515	1052	< 0.001	482	609	< 0.001	575	701	< 0.001	319	481	< 0.001
Mean of predicted probability of hospital birth (%)	33.2			32.7			26.6			29.6		

*EST* Estimate, *SD* Standard error, *EDF* Estimated degrees of freedom, *REF DF* Reference degrees of freedom

**Fig. 2:**
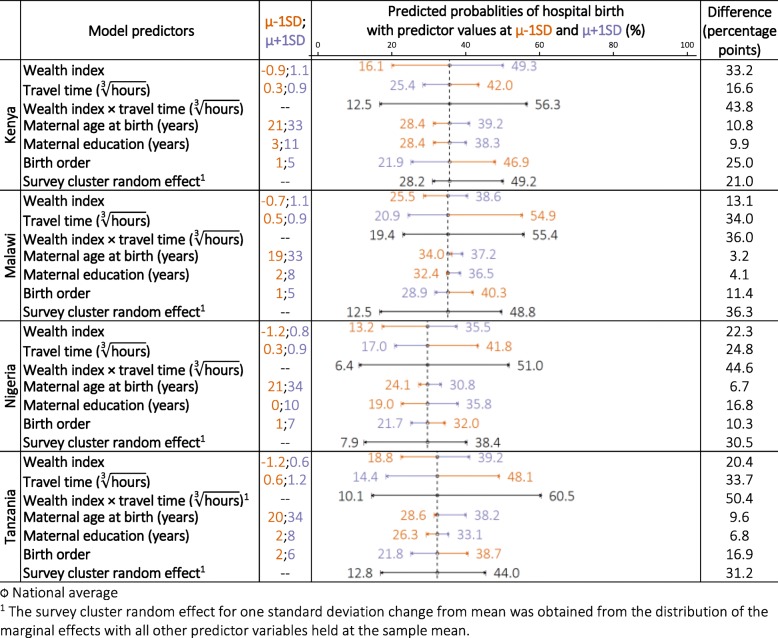
Marginal effects of one standard deviation (SD) change from mean (μ) of the predictor variables on the predicted probabilities of hospital birth

Figure 2 shows the marginal effect of 1 SD change from mean for each predictor variable whilst holding other model covariates at sample mean. In Kenya, compared to the average model-predicted value of 33.2%, a decrease in wealth index by 1SD from the mean reduced the predicted probability of hospital birth to 16.1%, and an 1SD increase from mean brought the predicted probability of hospital birth to 49.3% – a difference of 33.2 percentage points between the 16th and 84th percentiles. The marginal effect of μ ± 1SD change for travel time was weaker than that of wealth index (16.6 percentage points). The overall additive effect between wealth index and travel time by 1SD around the mean was 43.8 percentage points. The marginal effect of μ ± 1SD change for maternal age at birth, maternal education and birth order were 10.8, 9.9 and 25.0 percentage points, respectively. Lastly, the survey cluster random effect for 1SD change from mean was obtained from the distribution of predicted probabilities of hospital birth, whilst holding all other predictor variables at the sample mean. Comparing survey clusters 1SD below and above the mean led to a change of 21.0 percentage points in the predicted probability of hospital birth.

In Malawi, the marginal effect of 1SD change in wealth was weaker than that of travel time (13.1 versus 34.0 percentage points), and additive effect between wealth and travel time was not notably stronger (36.0 percentage points) than individual effect of travel time alone. In Nigeria, the marginal effects of wealth and travel time was similar (22.3 and 24.8 percentage points), and their additive effect was considerably stronger (44.6 percentage points). In Tanzania, the marginal effect of wealth was weaker than that of travel time (20.4 versus 33.7 percentage points), and their additive effect was stronger (50.4 percentage points). In all three countries, the marginal effects of maternal education, maternal age at birth and birth order were weaker than that of wealth and travel time. Survey clusters 1SD below and above the mean led to a change of approximately 30 percentage points in the predicted probability of hospital birth.

### The additive effect of wealth and travel time

**Fig. 3:**
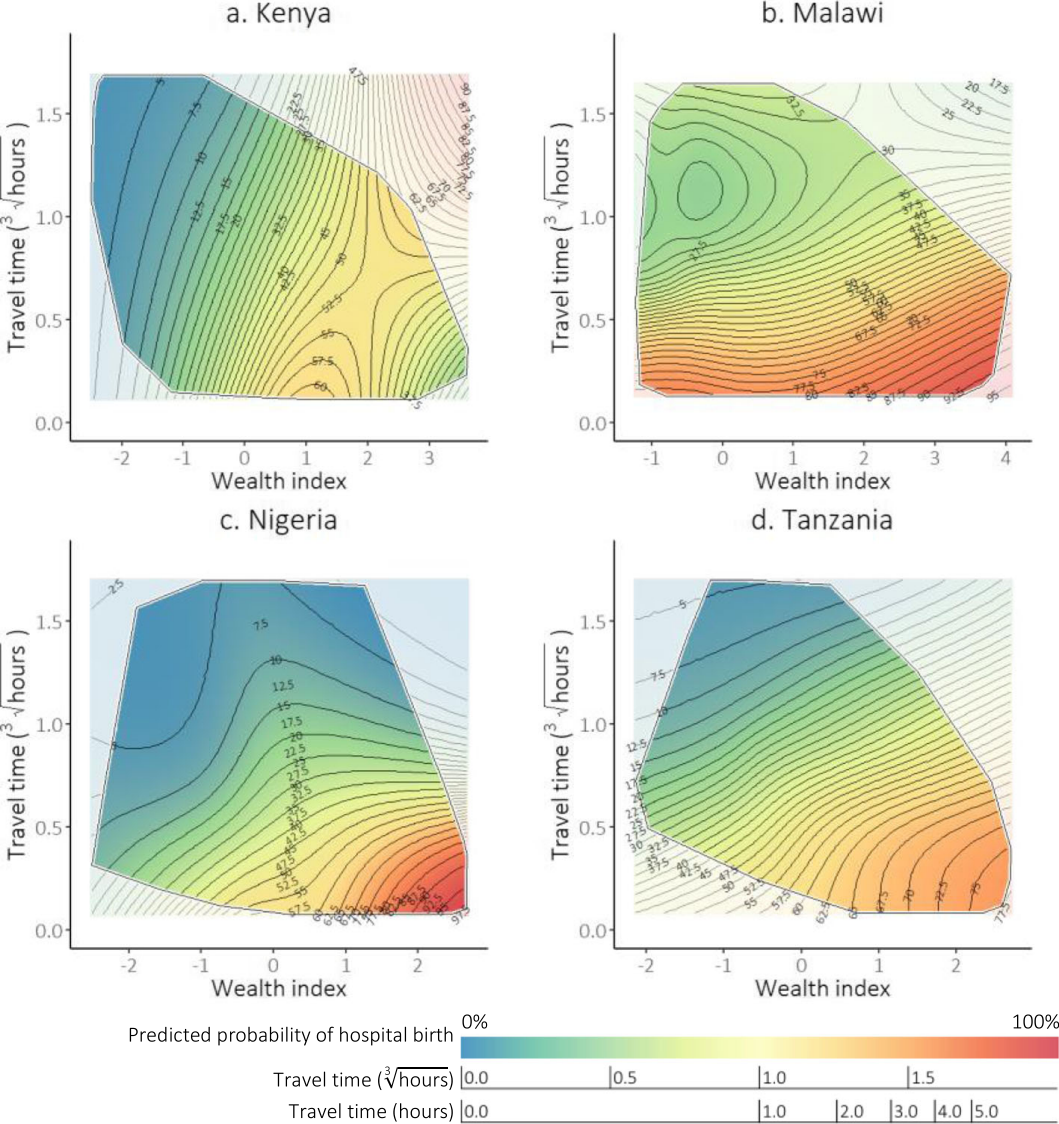
Predicted probability of hospital birth by travel time to the nearest hospital and household wealth index ^^^. ^^^ Model covariates – maternal education, maternal age at birth and birth order – were set to sample mean. Random effect at the survey cluster level was applied. All the observed combinations of values between travel time and wealth index were contained within the border. The colour gradient represents the value of the predicted probability of hospital birth (red: highest probabilities; blue: lowest probabilities). Contour lines are drawn to connect points that have the same predicted values. We drew contour lines for each 2.5% point increment in the predicted probabilities of hospital birth

We then plotted the additive effects between wealth and travel time as response surfaces, with the other model predictors held at the sample mean (Fig. 3). The response surfaces show the predicted probabilities as a function of travel time and wealth. In all four countries, livebirths to women who lived closer to a hospital and were from the least poor (lower right corner of the graph) had the greatest predicted probability of hospital birth; whilst the poorest who lived furthest away (top left corner) had the lowest. In Kenya, however, the predicted probability of hospital birth was low for the poorest, regardless of travel time. In addition, the increase in predicted probability of hospital birth with wealth index levelled off for the least poor. On average, in Malawi the predicted probability of hospital birth was high only for those living close to a hospital, regardless of wealth. In Nigeria, the predicted probability of hospital birth was low for those with either a long travel time or a low wealth index.

The angle of the contour lines represents the responsiveness of predicted probabilities of hospital birth to changes in the two predictor variables. Contour lines angled close to being vertical in Kenya show that the predicted probabilities of hospital birth were more responsive to changes in wealth, and the effect of travel time was relatively weaker – in line with results shown in Fig. 2. In Malawi, contour lines were angled more horizontally, indicating responsiveness of hospital birth to changes in travel time. In Nigeria, hospital birth was most responsive to changes in travel time among those who were far and poor, and less so for those who were far but less poor. The predicted probabilities of hospital birth were more responsive to changes in travel time for those living very far away in Tanzania.

The spaces between contour lines are widest among those who have the lowest predicted probability of hospital birth in Kenya, Nigeria and Tanzania, thus for them a fixed unit decrease in travel time and a fixed unit increase in wealth would have the smallest effect on the outcome. In Malawi, on the other hand, the widest gaps between contour lines were among those who have the highest predicted probability of hospital birth, for whom decreasing travel time or improving wealth would have the smallest increase in the likelihood of such births.

### GAMs residuals

**Fig. 4:**
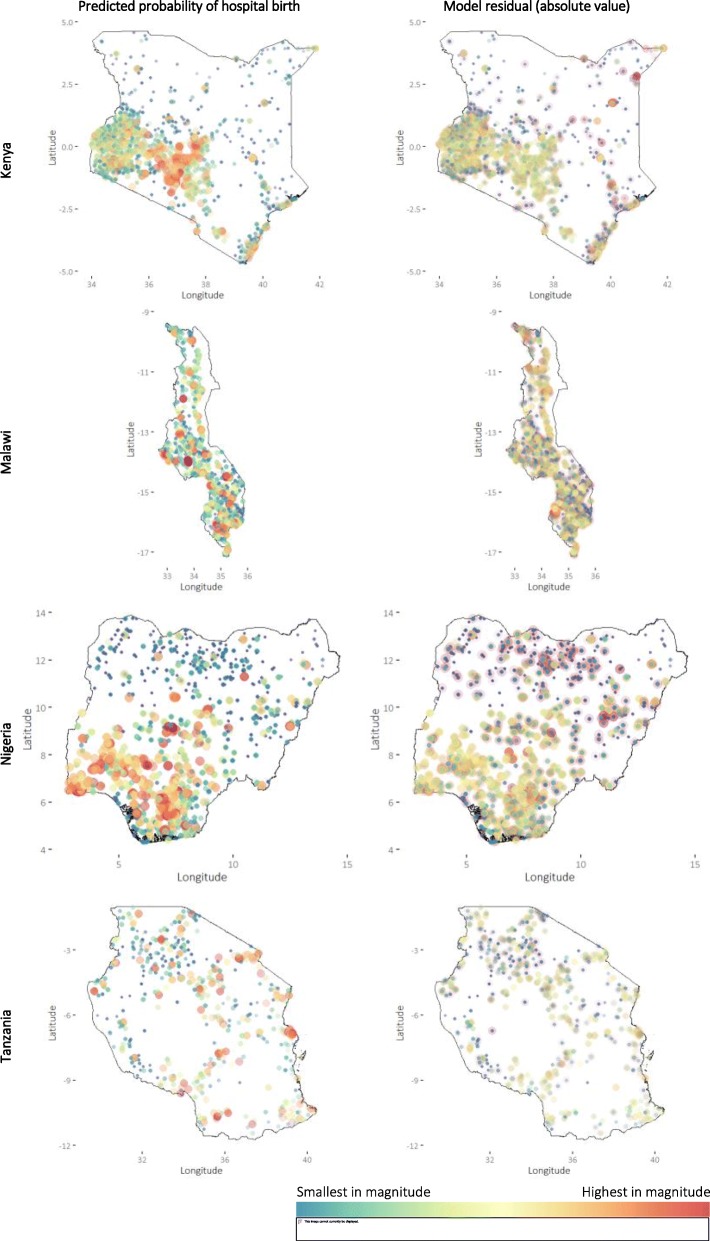
Model predicted probabilities of hospital birth and model residuals

Model residuals can show the extent of the variance in the data not explained by the model, with higher values indicating worse model fit. Model residuals were generally smallest when the predicted probability of hospital birth was low (Fig. 4), estimated travel time was short and wealth index was low to medium (Additional file 1). But there are exceptions; some groups of DHS clusters with low-to-medium predicted values stand out with large residuals, such as in Elwak, Bella Wagberi and Zubak in Kenya, Lilongwe in Malawi, and Kano and Gombe in Nigeria. In Nigeria, both high proportion of predicted hospital birth and high model residuals were mostly in the south, except for some costal clusters in southern Delta and Bayelsa States along the Gulf of Guinea.

## Discussion

### Summary of study results

Poverty and long travel time to health services are important barriers of maternity care-seeking in LMICs. They are commonly treated as collinear, and their separate effects have not been studied extensively. To our knowledge, this is the first study to partition their effects on hospital-based childbirth. We confirmed the substantial barriers posed by poverty and long travel time in Kenya, Malawi, Nigeria and Tanzania. By separating the effects of poverty and travel time, we found that the situation differed by country. The marginal effect of wealth on hospital birth was stronger than that of travel time in Kenya; the opposite was observed in Malawi and Tanzania. In Nigeria, the two were similar but their additive effect was twice as influential as their separate effects. Also, in Nigeria, hospital birth was generally most responsive to changes in travel time for women who were poor and lived the furthest away from a hospital. In most cases, women who were already least likely to give birth in a hospital would benefit the least from changes in wealth and travel time. Although both poverty and travel time were important, the random effects of survey clusters explained a substantial extent of between-cluster variability in hospital birth in all countries, indicating other unobserved local factors were at play.

### Interpretation of results

The differences in the relative contribution of poverty and long travel time on giving birth in a hospital within and across countries identified in our results require a context-specific interpretation. In Kenya, we found that wealth index was the predominant determinant of hospital birth for those from low- and middle-SES households. The Kenyan governments has implemented various pro-poor interventions to support the use of maternal health services since the early 2000 – including childbirth fees abolishment in 2007 in government dispensaries and health centres (with the replacement of a registration fee of 10–20 Kenyan Shillings, ≈ 0.1–0.2 US dollars) [39, 40], and from 2006 to 2016 a reproductive health voucher programme under which poor women could purchase subsidized vouchers for 200 Kenyan Shillings to cover the cost of antenatal care, facility childbirth and postnatal care [41, 42]. In 2013, the government extended the abolishment of maternity services (including childbirth) fees in all levels of government health facilities under the Free Maternity Services (FMS) policy [43]. Data used in our analysis primarily included childbirth prior to this change; other studies conducted afterwards have shown positive overall results – including sustained increase in hospital-based childbirth (1–2 years post implementation) [44, 45], higher rates of childbirth in hospitals than in lower-level facilities [46], greater increase of childbirth than antenatal care in hospitals [47], and a mild decline in the use of low-cost private hospital for childbirth [47] – but a 2019 study found small gains in the wealth-inequality of skilled childbirth services following the announcement of the FMS policy due to a relatively small increase in service uptake among low SES women to catch up with existing inequality gap [48].

In Tanzania, where both the number of hospitals by land area and average travel time to the nearest hospital were the least optimal among countries studied here [14, 21], we found that the effect of travel time was greater than that of wealth. Hospitals in Tanzania are primarily located in the southern and northern regions, with lower-level facilities serving rural areas in the central region. The Tanzanian government is committed to expanding service coverage so that people “don’t have to travel long distance to access the services in distant facilities”, putting forward projects to adding and renovating government health facilities in recent health policy plans [49, 50]. Both the Kenyan and Tanzanian governments have shown commendable attempts to support the use of maternal healthcare (including for childbirth) by removing user fees in public health facilities (Kenya and Tanzania) and making services geographically closer to the population (Tanzania) [49, 50]. The implementation of these different strategies, however, seems to face similar challenges. In Kenya, limited pre-existing health infrastructure and other supply-side capacity to match the increased workload following fee removal, insufficient referral and emergency obstetric care capacities contribute to persisting poor maternal (and newborn) health and its inequalities [51, 52]. Indeed, decline in maternal/neonatal mortality and stillbirths does not appear to have followed as a result of increase in facility utilization for childbirth [44, 53]. FMS in Kenyan government facilities may also have limited impact on increasing hospital birth for the poorest and the most remote women/families (among whom mortality and morbidity are typically the highest) due to the small number of hospitals that are within their reach [52]. For Tanzania, some studies suggest that policies aiming to reduce distance or travel time, by expanding service provision, deteriorate service quality when scarce resources are diluted. This may put the poorest people who cannot pay the cost of bypassing their nearest facility at higher risk of receiving suboptimal care [54, 55]. To ensure access to adequate care for all, concerted effort and innovative targeting are required. In a setting of high facility density and limited resources, it has been shown that concentrating available resources in fewer, but strategically selected, facilities/sites may promote geographic accessibility for all. In Tanzania and other LMICs, positive outcomes in physical accessibility and quality of care were achieved when interventions were supported by the right tools and approaches [55–58].

The government of Malawi promotes childbirth at primary health facilities, with referral to hospitals for women known to be at high risk [59, 60]. As part of the Banda era legacy, Malawi had a reasonably strong health centre system, and in a relatively well populated small rural country this meant that most women were not geographically too far from one of these facilities. Health services in the government sector are free-of-charge at the point of use in the country [59]. Since 2006, the government has also been progressively exempting childbirth fees for catchment populations of Christian Health Association of Malawi (CHAM) health facilities (often located in remote areas; approximately 40 and 25% of hospitals and health centres in the country are CHAM facilities, respectively [61]). Malawi has attained a near universal level of facility birth – 91% of livebirths in the 5 years before the 2015/16 DHS were delivered in a health facility [62] – yet only an estimated 25% of obstetric complications occurred in facilities with the capacity to provide the level of obstetric and newborn care required (such as in a hospital) [63, 64]. In pre-hospital settings, the median distance to the nearest point of obstetric surgical care is over 30 km. In *The Lancet*’s Maternal Health series in 2016, Campbell and colleagues called for all women to give birth in health facilities that can guarantee at least basic emergency obstetric care standard and timely referral for women with complications to higher-level care to ensure safe motherhood [1]. Our results suggested that the overall effect of travel time on hospital birth was greater than that of wealth, and their additive effect did not substantially explain further variability. Measures should be put in place to improve physical accessibility to EmONC services, including strengthening the capacity of health centres (to which some solutions are available to strategically select locations for facility upgrading that balances travel time across the whole population and equity as defined by wealth subgroups [14]); and expanding the provision of free maternal healthcare at more CHAM hospitals, especially those that are in very remote locales. However, recent reduction of development partners’ contribution to the Malawian total health budget has impaired the fee exemption mechanism with CHAM, resulting in certain facilities re-introducing user fees to cope with the financial setback. Such reduction is speculated to be related to internal political instability, scandals and poor governances [59, 65]. Strategies that include fee-based, non-profitable health providers working in rural areas mitigates financial barriers to use of care and expands the options for higher-level health providers that poor remote dwellers are otherwise unable to use, thus shortening the travel time required to obtain and receive adequate care [66, 67]. Long-term implementation of these strategies should not be hampered by unfavourable policy environment and government challenges.

In Nigeria, women who either had to travel for long or were poor were very unlikely to give birth in a hospital. These women were concentrated in specific geographic settings, with the poorest being largely in the north, and especially in Yobe State, while women travelling for long were mostly in the southern coastal areas in Delta and Bayelsa States. For those in Yobe State, the effect of travel time appeared to be very strong. The state has one of the lowest levels of skilled care for childbirth in the country [68], and while several studies have found ethnicity, social norm and religion as fundamental reasons for homebirths, there were also very few health facilities in the region [69]. Lembani and colleagues further posited that the Boko Haram Insurgency in the area since 2011 has resulted in the destruction and closing of many health facilities, with health personnel preferring to relocate in other areas [68]. The general lack of service provision in the area may have strongly affected the population’s ability to access health services. On the other hand, for those in the south who are approximately equally far but are relatively less poor, wealth played a relatively stronger role. Difficult riverine terrains in Bayelsa State pose additional impediments to overcoming travel-related barriers [70]. Although the area’s energy sector has generated interest among multi-national companies [71], most Bayelsans remain poor, while the state’s public infrastructure is underdeveloped [72, 73]. The proportion of women in Bayelsa who cited financial reasons for homebirth is higher than the national average [74]. Under such special economic and environment conditions, wealth may be additionally helpful for overcoming cost of transport, as well as trade-offs in time and financial loss from daily/productive activities.

In the context of health equity, horizontal equity refers to the principle that people with the same needs should have a similar level of access to the required health services; this contrasts to vertical equity which denotes unequal access to healthcare for people with different needs [75–77]. Assuming the need for skilled and adequate care for childbirth is universal or somewhat even across all population subgroups by sociodemographic characteristics (e.g., wealth and place of residence), the principle of horizontal equity is met if service uptake is also similarly distributed. In many LMICs, however, this is not the case. Wealth and physical accessibility to care continue to act as drivers of inequitable uptake of health services. Understanding variability in hospital births by poverty and travel time is useful for the design of policies to reduce inequity and strategies to reach populations that are likely to be left behind in terms of access to services. In addition, we note that our analysis revealed substantial survey cluster random effects, demonstrating local factors other than wealth and travel time are at play, and may limit the impact of strategies that are aimed at removing financial and accessibility barriers. Future studies are required to identify such local factors and how they can be overcome.

### Study limitations

Our results have important implications but should be interpreted with a few limitations in mind. First, the estimation of travel time from DHS cluster centroids to the nearest hospital using the MAP friction surface assumes a generalized travel speed by the type of land surface, and does not account for variabilities in temporality, seasonality and transportation used by the individuals. In particular, in rural areas characterized by a high level of poverty, walking and non-motorized vehicles remain the major means of transportation, while adoption of motorized transportation is limited by affordability issues [78–81]. In contrast, there is a wider range of transportation in urban settings. Of these, private and privately-owned vehicles – such as matatus in Kenya – have become very common. In poorer urban areas, however, many people still struggle to afford the fees to take these private vehicles and walk, whilst others who can afford them face challenges due to poor road networks [82–84]. The additional cost, time and difficulty of movement likely mean that we may have underestimated travel time for urban poor households, and the true negative effect of long travel time on hospital-based childbirth may be stronger than the effect estimated. Second, the accuracy of our estimates of the effect of travel time may be influenced by the displacement of DHS cluster. Applying Karra and Canning’s proposed method to correct the biased estimator with the expected minimum distance [85], Sato and colleagues found larger corrected effects than the uncorrected effects for distance on facility-based childbirth and attendance by doctor in Tanzania, although the differences were small (< 2 percentage points) [86]. Third, we excluded DHS clusters for which the estimated travel time from the MAP friction surface was over 5 h. In checking our travel time estimates against those obtained from OSM Routing Services, larger discrepancies tended to come from long travel time estimates using the MAP friction surface. This only affected a small number of data points (12 in Kenya, 6 in Tanzania and none in Malawi and Nigeria), but more detailed validity assessment of travel time estimates might be relevant in future work where manual checking becomes a feasible task. Fourth, this analysis employed data on livebirths in the 5 years preceding survey interviews and hospital data at given timespans. Although their occurrences are rare, we may have missed a very small number hospitals that may have been opened, closed, upgraded or downgraded between the time of survey and listing of facilities. Fifth, the use of wealth index as a measure of poverty may not accurately identify the very poor [7]; this may be particularly true for Malawi where the data appears to be considerably right-skewed. Sixth, we used one standard deviation around the mean as a consistent unit of change in our comparison of marginal effects of the model predictors. Other choices of unit (e.g. 5- or 10-year increment in maternal age at birth and maternal education, 60-min change in travel time) may vary the comparison and lead to different results. Last, our definition for hospital was based on data on the type of health facility as given in the master facility lists; the hospitals may vary in capacity, quality of care, and the range of health services that they provide. Such unmeasured covariates may confound the exposure to the outcome of our study.

## Conclusion

By assessing the relative contribution of poverty and long travel time, we found that these two factors determine whether women give birth in hospitals to a varying extent within and across the four study countries. For the poor and those living in remote areas who do not give birth in hospitals, the effect of poverty was stronger in some cases, while the effect of long travel time was stronger in others. Given the focus of “leaving no one behind” in the Universal Health Coverage agenda, more precise identification of population subgroups who are more likely to be left behind in terms of access to health services warrants further research. Such additional understanding can help inform the financial and geographic barriers that people face, devise tailor-made system-wide strategies to bring skilled care to meet health needs, and ultimately contribute to attaining the desired improvements in maternal and newborn health in resource-limited settings.

## Supplementary information

**Additional file 1.** Supplementary A: Additional information on the travel time estimates. Supplementary B: Model ouput. Supplementary C: Model predictions, model residuals, travel time and wealth index.

## Data Availability

The datasets generated analysed during the current study are available in the following repositories: 1. dhsprogram.com 2. https://explorer.earthengine.google.com/#detail/Oxford%2FMAP%2Ffriction_surface_2015_v1_0 3. http://downloads.afyaresearch.org/mfl/AbridgedeHealth Kenya Facilities Sept 2015.xls 4. kmfhl.health.go.ke 5. https://databox.worldbank.org/en/dataset/nigeria-nmis-health-facility-data-2014 6. http://moh.go.tz/hfrportal/index.php?r=site/index
