# Supplementary material for: Too poor or too far? Partitioning the variability of hospital-based childbirth by poverty and travel time in Kenya, Malawi, Nigeria and Tanzania
Source: Int J Equity Health. 2020 Jan 28;19:15. doi: 10.1186/s12939-020-1123-y (PMC6988213; doi:10.1186/s12939-020-1123-y)
Supplement: Supplementary file 1 — Additional file 1. Supplementary A: Additional information on the travel time estimates. Supplementary B: Model ouput. Supplementary C: Model predictions, model residuals, travel time and wealth index. [file 12939_2020_1123_MOESM1_ESM.docx]

## Supplementary A: Additional information on the travel time estimates

**Checking estimates of travel time**

Travel time estimated from the friction surface and that obtained from the OpenStreetMap project via the osrm package in R are shown in Figure A.1. Pearson correlation coefficients of the two sets of estimates in all countries are above 0.75. This suggests good alignment of the two. Larger discrepancies between the two sets of estimates arise from longer travel time estimated using the friction surface, with low corresponding OSRM estimates. This is particularly pertinent in Kenya and Malawi.

We drew 2km and 5km buffer circles around urban and rural clusters, respectively. For each cluster, a random sample of ten points were selected within the buffer circle, and the travel time to the nearest hospital were obtained for each random point (example for DHSCLUST=1101 given in Figure X). The median of the ten shortest travel time estimates was taken as the final travel time for the cluster. OSRM server may not be able to find a route for the exact start and end points, in which case a proximal route would be returned.

| **Kenya (pcorr=0.78, p<0.001)** | **Malawi (pcorr=0.82, p<0.001)** |
| --- | --- |
| 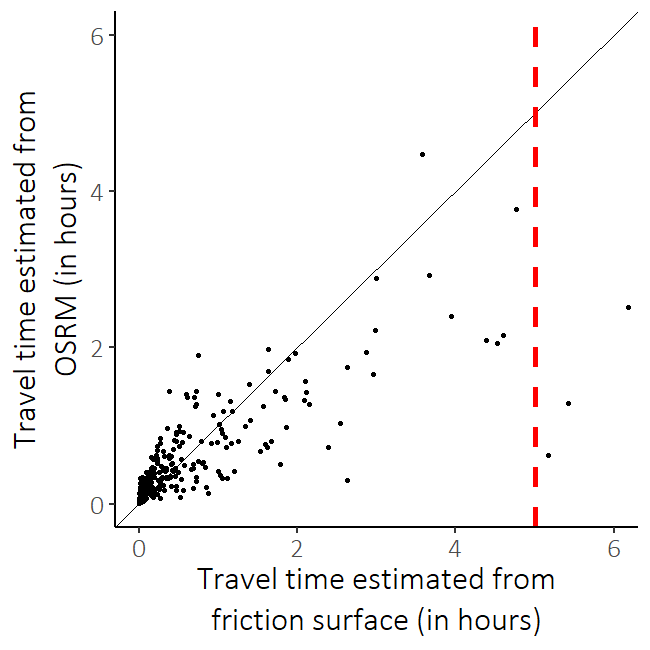 | 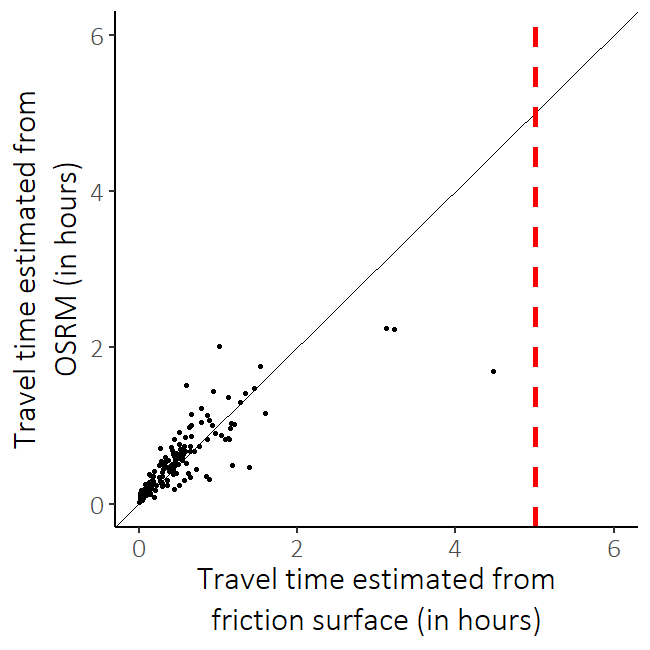 |
| **Nigeria (pcorr=0.80, p<0.001)** | **Tanzania (pcorr=0.87, p<0.001)** |
| 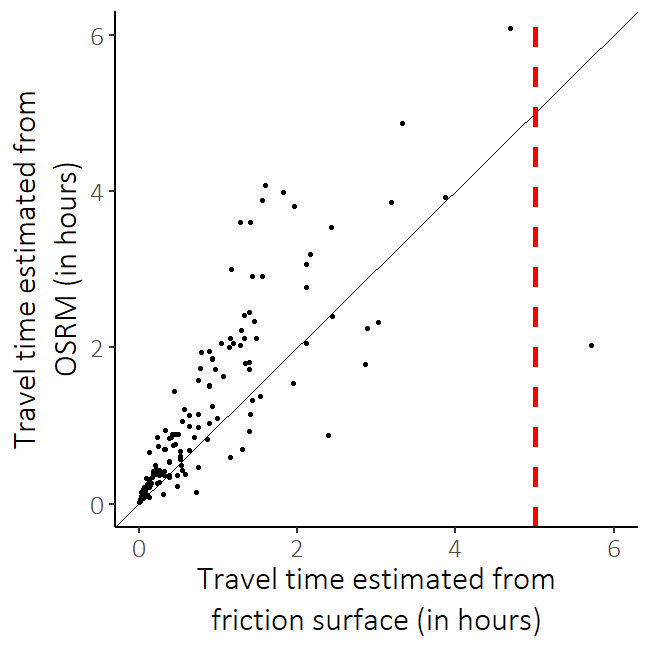 | 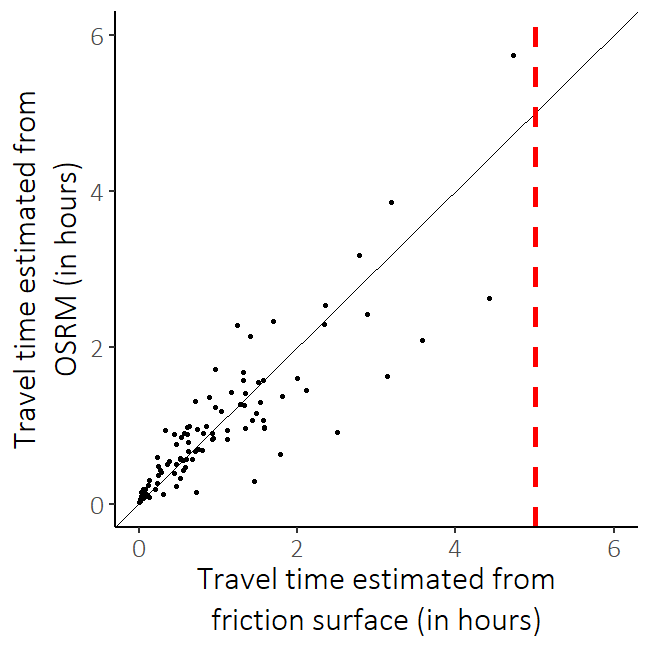 |

Note: due to a large number of hospitals in Nigeria, the same subset of hospitals were used for this check against OSM estimates.

| DHSCLUST | Fric.time | Osrm.time | %diff |
| --- | --- | --- | --- |
| 440 | 310 | 37 | 88 |
| 462 | 446 | 76 | 83 |
| 1036 | 325 | 69 | 79 |
| 1015 | 411 | 103 | 75 |
| 1094 | 405 | 113 | 72 |
| 485 | 432 | 140 | 68 |
| 1101 | 439 | 141 | 68 |
| 494 | 371 | 158 | 57 |

Estimated travel time to the nearest hospital using land surface friction and OSRM routes


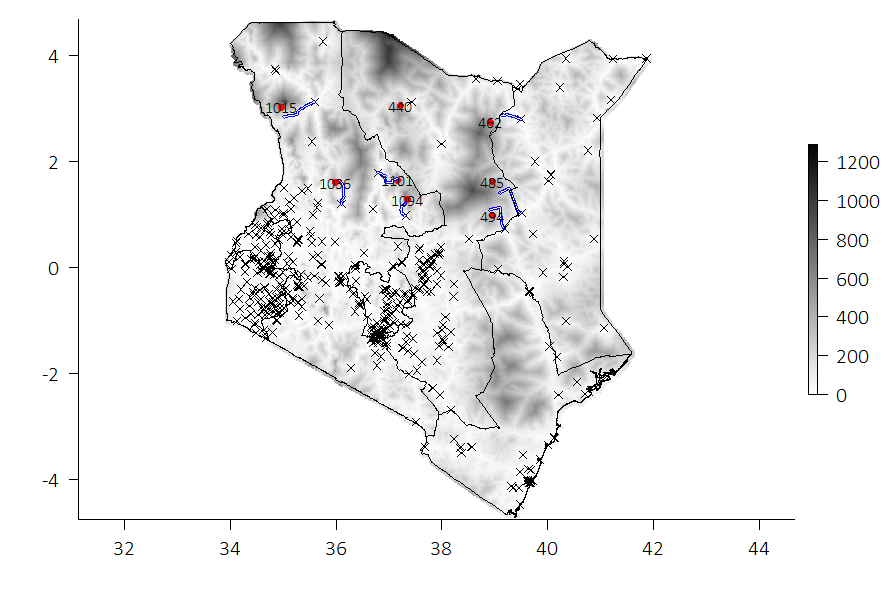

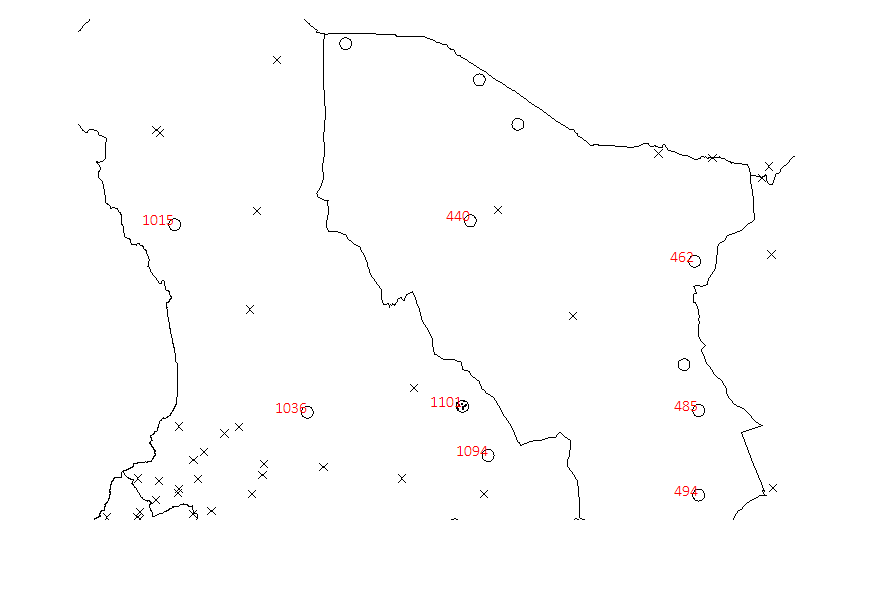


**Supplementary B: Model ouput**

Table B1. Deviance explained (DE) and %DE of different model formulations

| **Structure of random effects** | | **DHS clusters only** | | | |
| --- | --- | --- | --- | --- | --- |
| **k used for travel time × wealth index^a^** | | **k=5** | | **k=10** | |
|  | | **DE^b^** | **% DE^c^** | **DE^b^** | **% DE^c^** |
| **Kenya** | **null** | 21958 | -- | -- | -- |
|  | **travel time** | 1388 | 6.3 | -- | -- |
|  | **wealth index** | 2659 | 12.1 | -- | -- |
|  | **travel time × wealth index** | 2780 | 12.7 | 2787 | 12.7 |
|  | **travel time × wealth index + covariates^d^** | 3435 | 15.5 | 3441 | 15.7 |
| **Malawi** | **null** | 15472 | -- | -- | -- |
|  | **travel time** | 1299 | 8.4 | -- | -- |
|  | **wealth index** | 645 | 4.2 | -- | -- |
|  | **travel time × wealth index** | 1443 | 9.3 | 1445 | 9.3 |
|  | **travel time × wealth index + covariates^d^** | 1560 | 10.1 | 1561 | 10.1 |
| **Nigeria** | **null** | 37383 | -- | -- | -- |
|  | **travel time** | 5483 | 14.7 | -- | -- |
|  | **wealth index** | 8785 | 23.5 | -- | -- |
|  | **travel time × wealth index** | 9157 | 24.5 | 9178 | 24.5 |
|  | **travel time × wealth index + covariates^d^** | 10136 | 27.1 | 10151 | 27.2 |
| **Tanzania** | **null** | 9985.6 | -- | -- | -- |
|  | **travel time** | 1633.8 | 16.4 | -- | -- |
|  | **wealth index21.0** | 1439.4 | 14.4 | -- | -- |
|  | **travel time × wealth index** | 1889.7 | 18.9 | 1895.7 | 19.0 |
|  | **travel time × wealth index + covariates^d^** | 2096.6 | 21.0 | 2080.8 | 20.8 |

^a^ k = number of knots used on the smoothed term for the travel time × wealth interaction

^b^ Deviance explained (DE) = DN – DR, where DN is the null deviance and DR = Σ[residuals(MODEL, type=”deviance”)^2^]

^c^ %DE = DE * 100/DN, where DN is the null deviance.

^d^ Model covariates were maternal education, maternal age at birth and birth order.

**Kenya**

> summary(gam)

Family: quasibinomial

Link function: logit

Formula:

hosp ~ eduyears + s(ageatbirth) + bord + te(timecube, v191, k = 5) +

s(v001, bs = "re")

Parametric coefficients:

Estimate Std. Error t value Pr(>|t|)

(Intercept) -0.182297 0.107594 -1.694 0.0902 .

eduyears 0.064933 0.009418 6.894 5.62e-12 ***

bord -0.281132 0.017320 -16.231 < 2e-16 ***

---

Signif. codes: 0 ‘***’ 0.001 ‘**’ 0.01 ‘*’ 0.05 ‘.’ 0.1 ‘ ’ 1

Approximate significance of smooth terms:

edf Ref.df F p-value

s(ageatbirth) 2.356 2.963 28.950 <2e-16 ***

te(timecube,v191) 6.479 7.306 83.953 <2e-16 ***

s(v001) 514.876 1052.000 1.482 <2e-16 ***

---

Signif. codes: 0 ‘***’ 0.001 ‘**’ 0.01 ‘*’ 0.05 ‘.’ 0.1 ‘ ’ 1

R-sq.(adj) = 0.305 Deviance explained = 26.8%

-REML = 7062.2 Scale est. = 1.048 n = 15585

> gam.check(gam)

Method: REML Optimizer: outer newton

full convergence after 6 iterations.

Gradient range [-0.00212677,0.001021191]

(score 7062.243 & scale 1.048045).

Hessian positive definite, eigenvalue range [0.002112653,5803.807].

Model rank = 1532 / 1532

Basis dimension (k) checking results. Low p-value (k-index<1) may

indicate that k is too low, especially if edf is close to k'.

k' edf k-index p-value

s(ageatbirth) 9.00 2.36 1.00 0.62

te(timecube,v191) 24.00 6.48 0.97 0.01 **

s(v001) 1496.00 514.88 NA NA

---

Signif. codes: 0 ‘***’ 0.001 ‘**’ 0.01 ‘*’ 0.05 ‘.’ 0.1 ‘ ’ 1

**Malawi**

> summary(gam)

Family: quasibinomial

Link function: logit

Formula:

hosp ~ eduyears + s(ageatbirth, bs = "cs") + bord + te(timecube,

v191, k = 5, bs = "cs") + s(v001, bs = "re")

Parametric coefficients:

Estimate Std. Error t value Pr(>|t|)

(Intercept) -0.551906 0.109263 -5.051 4.45e-07 ***

eduyears 0.028308 0.009371 3.021 0.00253 **

bord -0.120546 0.021231 -5.678 1.39e-08 ***

---

Signif. codes: 0 ‘***’ 0.001 ‘**’ 0.01 ‘*’ 0.05 ‘.’ 0.1 ‘ ’ 1

Approximate significance of smooth terms:

edf Ref.df F p-value

s(ageatbirth) 2.885 9 4.368 4.99e-06 ***

te(timecube,v191) 10.707 24 306.642 < 2e-16 ***

s(v001) 482.247 609 4.064 < 2e-16 ***

---

Signif. codes: 0 ‘***’ 0.001 ‘**’ 0.01 ‘*’ 0.05 ‘.’ 0.1 ‘ ’ 1

R-sq.(adj) = 0.333 Deviance explained = 30.5%

-REML = 5940.8 Scale est. = 1.0127 n = 14047

> gam.check(gam)

Method: REML Optimizer: outer newton

full convergence after 7 iterations.

Gradient range [-0.004679196,0.004154062]

(score 5940.844 & scale 1.012713).

Hessian positive definite, eigenvalue range [0.4965591,5450.065].

Model rank = 864 / 864

Basis dimension (k) checking results. Low p-value (k-index<1) may

indicate that k is too low, especially if edf is close to k'.

k' edf k-index p-value

s(ageatbirth) 9.00 2.88 1.00 0.55

te(timecube,v191) 24.00 10.71 1.01 0.90

s(v001) 828.00 482.25 NA NA

**Nigeria**

> summary(gam)

Family: quasibinomial

Link function: logit

Formula:

hosp ~ eduyears + s(ageatbirth, bs = "cs") + bord + te(timecube,

v191, k = 5, bs = "cs") + s(v001, bs = "re")

Parametric coefficients:

Estimate Std. Error t value Pr(>|t|)

(Intercept) -1.563824 0.079401 -19.695 <2e-16 ***

eduyears 0.086800 0.004891 17.745 <2e-16 ***

bord -0.103559 0.011888 -8.711 <2e-16 ***

---

Signif. codes: 0 ‘***’ 0.001 ‘**’ 0.01 ‘*’ 0.05 ‘.’ 0.1 ‘ ’ 1

Approximate significance of smooth terms:

edf Ref.df F p-value

s(ageatbirth) 2.539 9 13.406 1.57e-11 ***

te(timecube,v191) 11.773 24 1023.667 < 2e-16 ***

s(v001) 574.575 701 4.616 < 2e-16 ***

---

Signif. codes: 0 ‘***’ 0.001 ‘**’ 0.01 ‘*’ 0.05 ‘.’ 0.1 ‘ ’ 1

R-sq.(adj) = 0.434 Deviance explained = 41.1%

-REML = 9617.1 Scale est. = 1.0181 n = 31208

> gam.check(gam)

Method: REML Optimizer: outer newton

full convergence after 7 iterations.

Gradient range [-0.002203074,0.001675076]

(score 9617.06 & scale 1.018132).

Hessian positive definite, eigenvalue range [1.053828,12668.04].

Model rank = 925 / 925

Basis dimension (k) checking results. Low p-value (k-index<1) may

indicate that k is too low, especially if edf is close to k'.

k' edf k-index p-value

s(ageatbirth) 9.00 2.54 0.99 0.46

te(timecube,v191) 24.00 11.77 0.96 0.01 **

s(v001) 889.00 574.58 NA NA

---

Signif. codes: 0 ‘***’ 0.001 ‘**’ 0.01 ‘*’ 0.05 ‘.’ 0.1 ‘ ’ 1

**Tanzania**

> summary(gam)

Family: quasibinomial

Link function: logit

Formula:

hosp ~ eduyears + s(ageatbirth, bs = "cs") + bord + te(timecube,

v191, k = 5, bs = "cs") + s(v001, bs = "re")

Parametric coefficients:

Estimate Std. Error t value Pr(>|t|)

(Intercept) -0.79500 0.14563 -5.459 4.96e-08 ***

eduyears 0.05399 0.01145 4.716 2.46e-06 ***

bord -0.16420 0.02799 -5.866 4.68e-09 ***

---

Signif. codes: 0 ‘***’ 0.001 ‘**’ 0.01 ‘*’ 0.05 ‘.’ 0.1 ‘ ’ 1

Approximate significance of smooth terms:

edf Ref.df F p-value

s(ageatbirth) 3.791 9 4.985 4.01e-06 ***

te(timecube,v191) 8.373 24 145.732 < 2e-16 ***

s(v001) 318.895 481 1.899 < 2e-16 ***

---

Signif. codes: 0 ‘***’ 0.001 ‘**’ 0.01 ‘*’ 0.05 ‘.’ 0.1 ‘ ’ 1

R-sq.(adj) = 0.374 Deviance explained = 35.5%

-REML = 3242.3 Scale est. = 1.2177 n = 7187

> gam.check(gam)

Method: REML Optimizer: outer newton

full convergence after 9 iterations.

Gradient range [-1.782754e-05,2.637179e-05]

(score 3242.262 & scale 1.217685).

Hessian positive definite, eigenvalue range [0.9157861,3285.951].

Model rank = 557 / 557

Basis dimension (k) checking results. Low p-value (k-index<1) may

indicate that k is too low, especially if edf is close to k'.

k' edf k-index p-value

s(ageatbirth) 9.00 3.79 0.98 0.135

te(timecube,v191) 24.00 8.37 0.96 0.005 **

s(v001) 521.00 318.90 NA NA

---

Signif. codes: 0 ‘***’ 0.001 ‘**’ 0.01 ‘*’ 0.05 ‘.’ 0.1 ‘ ’ 1

## Supplementary C: Model predictions, model residuals, travel time and wealth index

|  | **Predicted probability of hospital birth** | **Model residual (absolute value)** | $\sqrt[\text{3}]{\text{Travel time to the nearest hospital}}$ | | **Wealth index** |
| --- | --- | --- | --- | --- | --- |
| **Kenya** | 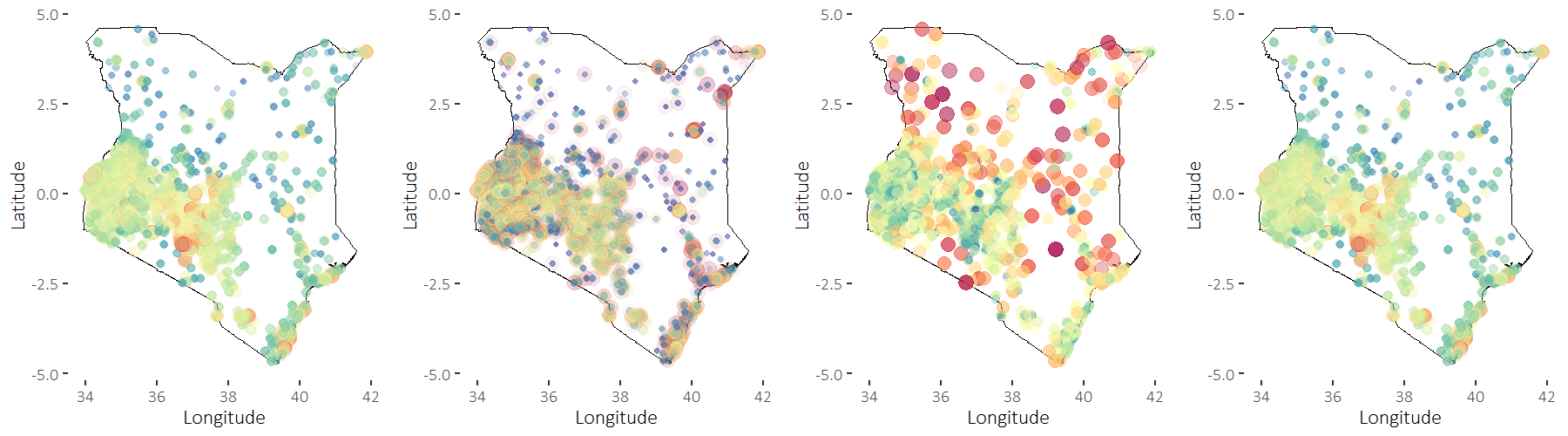 | | | | |
|  |  |  | Lowest | Highest | |
|  |  | | 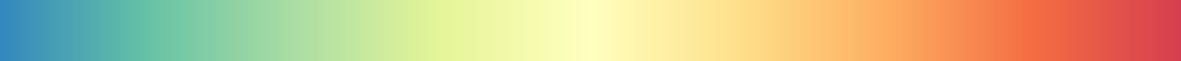 | | |

|  | **Predicted probability of hospital birth** | **Model residual (absolute value)** | $\sqrt[\text{3}]{\text{Travel time to the nearest hospital}}$ | | **Wealth index** |
| --- | --- | --- | --- | --- | --- |
| **Malawi** | 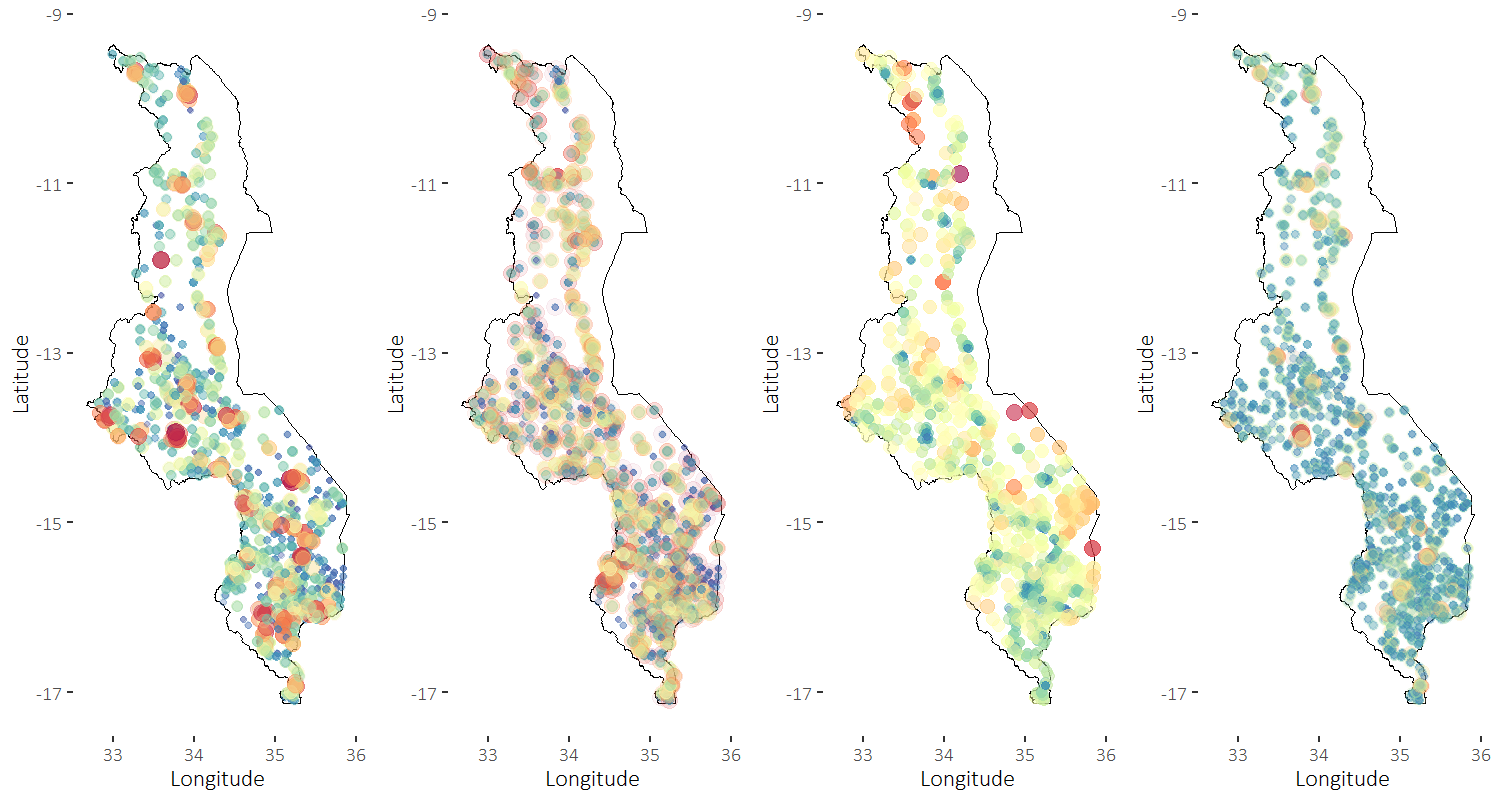 | | | | |
|  |  |  | Lowest | Highest | |
|  |  | | 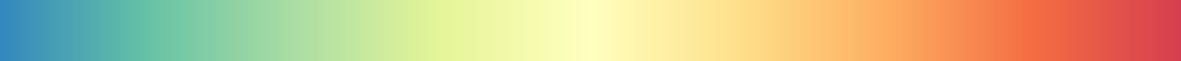 | | |

|  | **Model residual (absolute value)** | **Predicted probability of hospital birth** | $\sqrt[\text{3}]{\text{Travel time to the nearest hospital}}$ | | **Wealth index** |
| --- | --- | --- | --- | --- | --- |
| **Nigeria** | 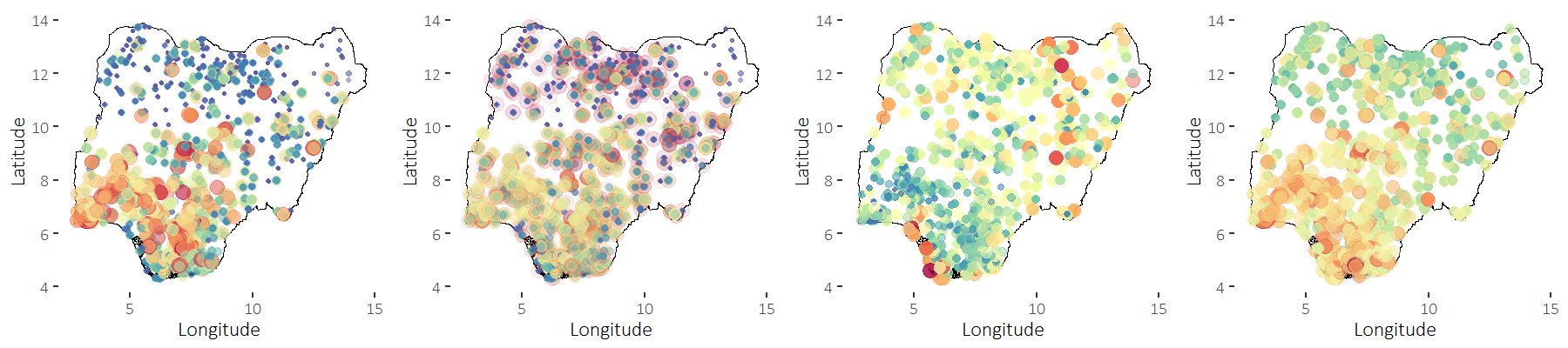 | | | | |
| **Tanzania** | 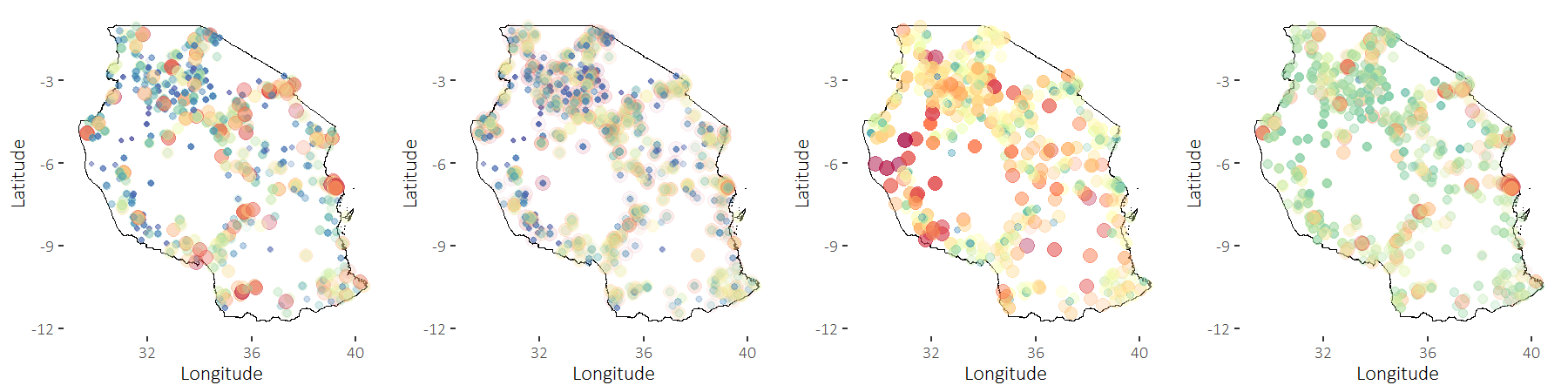 | | | | |
|  |  |  | Lowest | Highest | |
|  |  | | 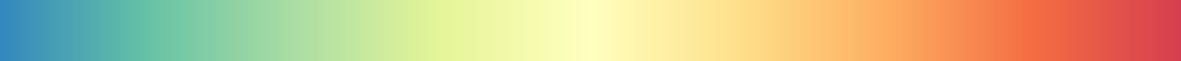 | | |
